# Head and Tibial Acceleration as a Function of Stride Frequency and Visual Feedback during Running

**DOI:** 10.1371/journal.pone.0157297

**Published:** 2016-06-07

**Authors:** Michael A. Busa, Jongil Lim, Richard E. A. van Emmerik, Joseph Hamill

**Affiliations:** 1 Biomechanics Laboratory, Department of Kinesiology, University of Massachusetts Amherst, Amherst, Massachusetts, United States of America; 2 Motor Control Laboratory, Department of Kinesiology, University of Massachusetts Amherst, Amherst, Massachusetts, United States of America; Harvard School of Dental Medicine, UNITED STATES

## Abstract

Individuals regulate the transmission of shock to the head during running at different stride frequencies although the consequences of this on head-gaze stability remain unclear. The purpose of this study was to examine if providing individuals with visual feedback of their head-gaze orientation impacts tibial and head accelerations, shock attenuation and head-gaze motion during preferred speed running at different stride frequencies. Fifteen strides from twelve recreational runners running on a treadmill at their preferred speed were collected during five stride frequencies (preferred, ±10% and ±20% of preferred) in two visual task conditions (with and without real-time visual feedback of head-gaze orientation). The main outcome measures were tibial and head peak accelerations assessed in the time and frequency domains, shock attenuation from tibia to head, and the magnitude and velocity of head-gaze motion. Decreasing stride frequency resulted in greater vertical accelerations of the tibia (p<0.01) during early stance and at the head (p<0.01) during early and late stance; however, for the impact portion the increase in head acceleration was only observed for the slowest stride frequency condition. Visual feedback resulted in reduced head acceleration magnitude (p<0.01) and integrated power spectral density in the frequency domain (p<0.01) in late stance, as well as overall of head-gaze motion (p<0.01). When running at preferred speed individuals were able to stabilize head acceleration within a wide range of stride frequencies; only at a stride frequency 20% below preferred did head acceleration increase. Furthermore, impact accelerations of the head and tibia appear to be solely a function of stride frequency as no differences were observed between feedback conditions. Increased visual task demands through head gaze feedback resulted in reductions in head accelerations in the active portion of stance and increased head-gaze stability.

## Introduction

Human locomotion has been described as self-optimizing in order to produce stable coordinated patterns that are also energy efficient [1, 2]. The optimization of these patterns affords the selection of safe and efficient paths of navigation [3–6]. Under steady state conditions without secondary tasks, individuals select movement patterns that minimize the amount of shock transferred to the head and are metabolically efficient. Specifically, modulation of stiffness [7], kinetics [8] and muscle activity [9] spanning the knee appear to be the mechanisms through which individuals modulate the amount of high-frequency shock transmitted to the head, and thereby possibly stabilize the visual field by reducing head motion [8, 10].

Investigations into walking have identified that individuals display stable head acceleration patterns across a wide range of step lengths, cadences, and speeds as well as a combination of stride lengths and cadences [11]. The stabilization of head accelerations across a range of speed and stride characteristics emerges through modification of the movement kinetics, kinematics and muscular activation patterns, and will afford a stable visual field and the identification of salient visual information and safe navigation through their environment [12].

Research on running mechanics has consistently manipulated stride frequency in order to investigate how changes in stride parameters impact the attenuation of shock and the regulation of head accelerations throughout the kinematic chain [8, 10, 13–15]. Specifically, Hamill et al. [10] found that individuals attenuate more of the high frequency impact shock at lower stride frequencies when running at preferred speed. This study identified that while running at preferred speed across a range of stride frequencies, spanning 20% below to 20% above preferred, individuals displayed near constant head accelerations. Additionally, they found that tibial accelerations are directly related to stride frequency such that tibial accelerations increased as stride frequency decreased (or stride length increased), but no differences in head accelerations were observed [10]. The attenuation of high frequency shock through the kinematic chain appears to emerge through changes at the knee, specifically, kinematics [7], kinetics [8], and muscle activity [9]. A common conclusion from existing research is that these changes in movement dynamics and shock attenuation serve to stabilize head motion and support visual information pick up; however, these claims have not been tested empirically as yet during running.

Fajen et al. [4] suggested that the perception-action coupling proposed by Gibson [16] is useful in the study of athletics. Specifically, examining the links between perception and action systems will allow for an improved understanding of how perceptual-motor patterns are selected and provide insight into the evaluation of performance tradeoffs. To date, many lines of research have evaluated the criteria from which individuals select preferred speed [17], and stride length and stride frequency [10, 18] that minimize metabolic cost of transport. Hamill et al. [10] suggested an alternative possibility, that individuals choose to adopt kinematic and kinetic patterns that support the stabilization of the visual field in addition to being metabolically efficient. Furthermore, when given an environment that requires a large amount of visual focus individuals would select stride patterns that prioritize visual field stability over metabolic economy. Specifically, low stride rates (or long stride lengths) are associated with increased muscle activity and stiffness of the knee and appear to be well suited to attenuate high frequency (>8 Hz) vertical accelerations associated with foot/ground impact [10, 19]. In general, the changes in gait mechanics that coincide with longer stride lengths appear well suited to dampen high-frequency shock. However, it should be noted that the lack of a visual task in these studies diminishes our ability to explicitly evaluate the contribution of perceptual factors to the observed changes in shock attenuation. Therefore, it is necessary to investigate tibial and head accelerations, the attenuation of shock to the head and head motion under visual tasks that place different demands on head motion dynamics.

The modulation of gait patterns to minimize shock transmission through the kinematic chain and regulate head acceleration is a critical factor in the successful navigation through complex environments [3, 5, 20]. Previous studies have shown that real-time visual [21] or auditory [22] feedback can reduce tibial accelerations. It is not clear, however, if the minimization of impact shock in response to the aforementioned feedback stimuli results in the anticipated changes in head accelerations. Providing real-time feedback of head-gaze motion while running at a range of stride frequencies at preferred speed will allow a direct assessment of how tibial and head accelerations and shock attenuation are modulated in responses to different visual tasks.

Therefore, the purpose of this study was to investigate if providing individuals with visual feedback of their head-gaze orientation impacts tibial and head accelerations, shock attenuation and head-gaze motion during preferred speed running at different stride frequencies. We hypothesized that regardless of visual feedback condition the impact shock at both the head and the tibia would be related to stride frequencies such that: 1) increased tibial and head accelerations would be observed for the portion of stance associated with foot-ground impact during running at low stride frequencies. This hypothesis was based on the results of previous research on running [8, 10, 13, 15]. Furthermore, we hypothesized that when provided with feedback of their head gaze orientation individuals would: 2) increase the amount of shock attenuated through the kinematic chain, and 3) reduce head accelerations in the portion of stance associated with active head control. These hypotheses were formed on the basis of suggestions from prior running research [10] as well as the findings of Latt et al. [11] on walking. In addition, we investigated to what degree providing individuals with visual feedback of their head-gaze motion would affect head gaze dynamics. We hypothesized 4) that providing head-gaze feedback would result in reduced head motion (amount and velocity) compared to when no feedback was given.

## Methods

### Participants

Twelve recreational runners (4 female, 8 male; 29.67 ± 4.4 years; 1.73 ± 0.08 m; 72.1 ± 13.9 kg, statistics combined for both genders) with a minimum preferred treadmill running speed of at least 2.3 m*s^-1^ volunteered to be participants in the study. All participants (preferred running speed 3.1 ± 0.39 m/s; range: 2.3–3.6 m/s) ran at least twice a week, were free of lower extremity injury for at least one year prior to testing, did not normally wear foot orthotics, and reported that they were free of any condition that would limit their ability to participate in the experiment. Prior to testing all participants filled out a Modified Physical Activity Readiness Questionnaire and a brief questionnaire regarding injury history, to ensure that they were physically capable of safely completing the testing protocol. All participants provided written informed consent, approved by the University Institutional Review Board before testing.

### Protocol

Once participants agreed to participate in the study, they were fitted with neutral racing flats (T7 Brooks, Seattle, WA, USA) to limit the effect of different midsole densities. Participants were then asked to identify their preferred treadmill running speed by directing the experimenter to increase or decrease belt speed until their preferred speed was identified [2]. This process was repeated until participants identified the same speed in successive trials, one where belt speed was gradually increased and another where it was gradually decreased. After preferred running speed was identified individuals were instructed to continue running at that speed while experimenters visually determined their preferred stride frequency.

**Fig 1:**
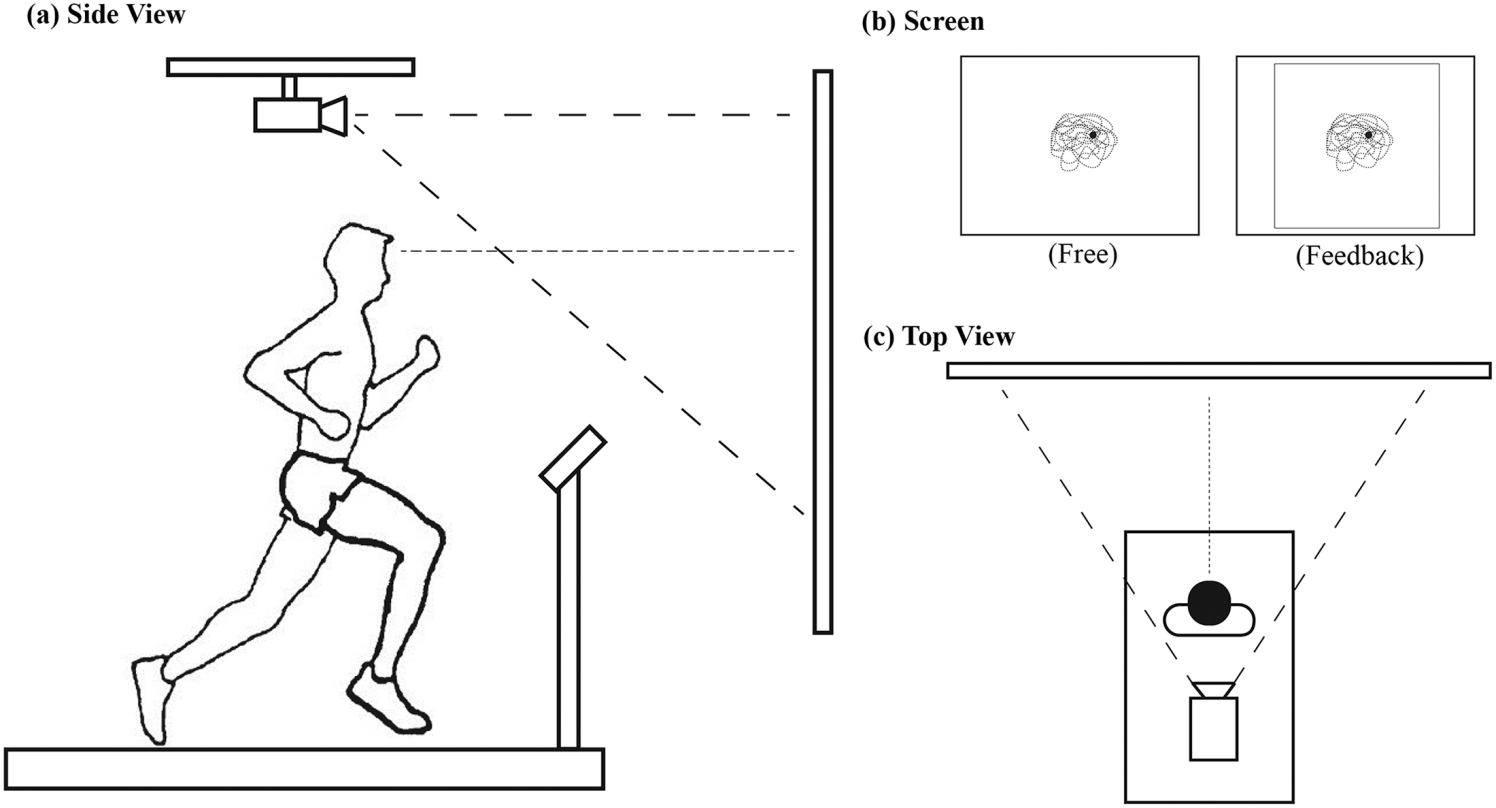
Experimental setup: side view (a) and top view (c). A screen was positioned approximately 2.5 m away from the treadmill center. A dotted line from the participant’s head in (a) and (c) indicates an imaginary line of a head gaze vector created from the 6-DOF object worn by participant. In the visual feedback condition (b-right), a white dot (colored black in b) indicating the intersection point of head gaze vector on the screen was displayed while running. In (b) the dotted line represents the trajectory of the head gaze point on the screen, this was not displayed during the testing. During the visual feedback condition (b-right) a square box (Inset light box) was displayed to indicate the boundary area for the feedback. This box was created by subtending an angle 21 degrees horizontally and vertically form the center of the treadmill centered at a height of 1.7m above the treadmill belt.

Next participants completed a series of short treadmill (StarTrac; Unisen, Inc., Irvine, CA, USA) runs at preferred speed in five stride frequency and two visual conditions. Stride conditions included preferred stride frequency (PSF), as well as frequencies corresponding to 80%, 90%, 110% and 120% of PSF (PSF-20, PSF-10, PSF+10, and PSF+20, respectively). At each experimental stride frequency condition, including preferred, participants ran while matching a preferred foot strike to the beat of an auditory metronome (Seiko Quartz, Seiko, Tokyo, Japan). This manipulation of stride frequency was used to vary stride length. Stride frequency conditions were randomized within each visual condition. Each of these stride frequency conditions was performed under two visual conditions: 1) a no feedback condition where participants were provided no information about their head gaze orientation; and 2) a visual feedback condition where participants’ real-time head gaze orientation was projected onto the same screen within a box of fixed dimensions. In both conditions, the instruction was the same: “run and match your footfalls to the metronome while looking forward at the screen”. In the condition without visual feedback, participants looked at a white screen in front of them (1.2 × 1.6 m). During the visual feedback condition, a 1.11 x 1.11 m box was projected on the screen in order to provide reference to the edges of the screen (Fig 1b). In addition, a dot representing the intersection point of head-gaze on the frontal plane was projected on the screen in real-time; this dot moved in real-time with the rotations and translations of the participant’s head (Fig 1b). The no feedback condition always performed before the visual feedback condition to eliminate the possibility of a carry-over effect. Thirty seconds of data from each combination of stride frequency and visual condition were collected.

### Experimental Apparatus

Qualisys Track Manager (Qualisys, Inc., Gothenburg, Sweden) was used to synchronize eight ProReflex cameras (Qualisys, Inc., Gothenburg, Sweden), and two triaxial accelerometers (Trigno; Delsys, Inc., Natick, MA, USA), operating at 120 and 1200 Hz, respectively. During the visual feedback condition, kinematic data of the head was imported in real time to a custom written Matlab program (The MathWorks, Inc., Natick, MA, USA) that was used to project a dot representing the intersection of the head gaze vector on a screen 2.5 m from the center of the treadmill (Fig 1).

A 3D biomechanical model of the left and right feet, right shank, right thigh, pelvis, trunk, and head was created from 42 reflective markers. Prior to testing, individuals performed two five second standing calibrations; the first was used to create the anatomical model of each participant; the second was used to capture the orientation of each participant’s head when looking directly at the center of the frontal visual plane.

Tibial and head accelerations were captured in the vertical, anterior-posterior and medial-lateral directions. Accelerometers were mounted to the anteromedial distal aspect of the right tibia and the frontal bone of the head [10, 23–25]. Each accelerometer was secured with strapping around both the head and lower legs; straps were tightened to the tolerance of each participant’s comfort in order to limit extraneous movement.

### Data Analysis

Time and frequency domain parameters from the tibial and head accelerometers were determined across 15 successive stance phases. Strides were identified from 3-D kinematic foot motion [26]. Tibial and head accelerations were detrended by subtracting a least-squares line of best fit from the raw data [19]. Accelerometer data were then low-pass filtered with a cutoff frequency of 60 Hz using a second order recursive Butterworth filter [27]. The power spectral density (PSD) of the head and tibial accelerations during stance was determined using a square window; frequency characteristics were normalized into 1 Hz bins [10]. Signal power magnitude in both the active and impact phases of stance were quantified by the integral of the signal power in the 3–8 Hz and 9–20 Hz frequency ranges, respectively.

For both the tibia and head the impact peak was identified as the peak acceleration occurring in 0–30% of stance and contained in the 9–20 Hz range in the PSD. The active acceleration peak was identified as the second peak in the head acceleration profile occurring between 31–100% of stance and in the 3–8 Hz range in the PSD [23]. Full procedures for processing the tibial and head accelerations are described in detail elsewhere [23]. The gain or attenuation of shock (i.e., the transmission of acceleration through the kinematic chain) from the tibia to the head was determined from a transfer function (Eq 1) which was used to calculate the PSD ratio of each frequency bin between the tibia and head signal [8, 10, 14, 28].

$$\text{Transfer Function }\left(\text{dB}\right)=10\times {\text{log}}_{10}\left({\text{PSD}}_{\text{Head}}/{\text{PSD}}_{\text{Tibia}}\right)$$ (1)

With PSD_Head_ and PSD_Tibia_ representing the PSD of the head and tibia, respectively. Positive values indicate a gain, or increase in signal strength at each frequency, and negative values indicate attenuation, or decrease in signal strength at each frequency. The magnitude of the gain or attenuation in the signal was quantified as the integral of the transfer function within the active (3–8 Hz) and impact (9–20 Hz) frequency ranges.

Head-gaze motion on the visual plane was assessed from the horizontal and vertical movements of the head gaze point on the frontal (target) plane that arise from the translations and rotations of the head in space (see Fig 1). Head-gaze performance in both visual conditions was evaluated by calculating the 95% ellipse area, path length and average velocity of the gaze path, as well as the range of motion of both the horizontal and vertical gaze point.

### Statistical Analysis

The effects of stride frequency and visual task on tibial and head accelerations, transfer function, and head gaze point dynamics were evaluated with a series of linear mixed model analyses of variance with visual task and stride frequency as fixed factors. Least squared difference *post hoc* tests were used when appropriate. All statistical testing was performed in PASW 18 (SPSS, Inc., Chicago, IL, USA). Significant differences were identified by an α < 0.05. Effect sizes were determined using partial eta-squared ($\eta_{p}^{2}$).

## Results

No interaction effects were observed between stride frequency and visual feedback conditions for any of the dependent variables (p-values > 0.05).

### Stride Frequency

As anticipated, the stride frequency differed significantly between the five different stride frequency conditions [F(4, 99) = 589.096, p<0.001, $\eta_{p}^{2}$ = 0.966]. Pairwise post-hoc comparisons showed all stride frequency conditions differed from one another [p<0.001, for all comparisons]. Additionally, the measured stride frequency did not differ between the visual feedback conditions [F(1, 99) = 2.347, p = 0.129, $\eta_{p}^{2}$ = 0.360].

### Tibial and Head Accelerations

**Fig 2:**
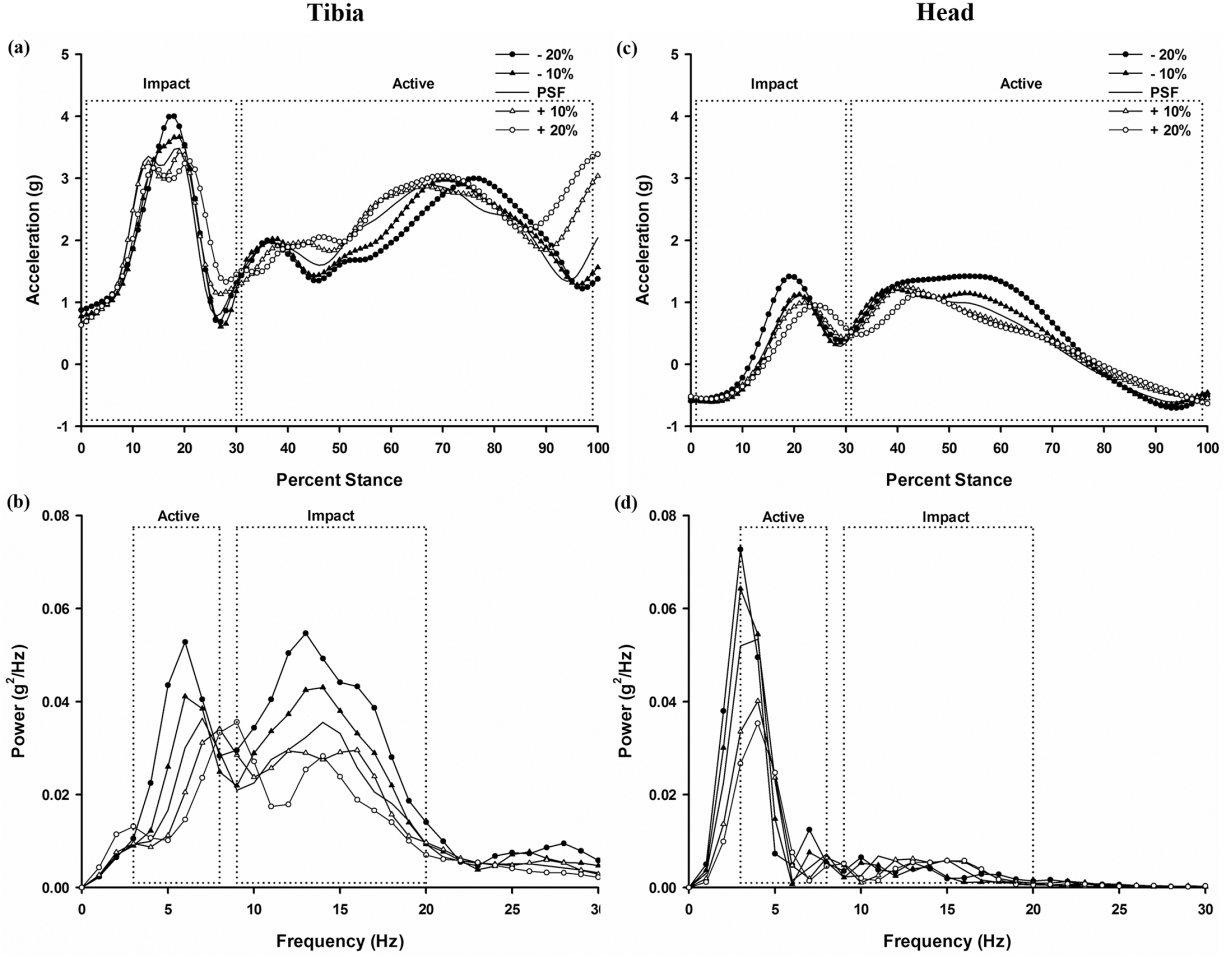
Tibial and head acceleration profiles during stance as a function of stride frequency. Representative participant data: tibia and head acceleration characteristic profile in time ((a) and (c)) and frequency ((b) and (d)) domain during a stance phase as a function of stride frequency.

Main effects of stride condition were observed for the tibia during the impact portion of stance for both peak acceleration [F(4, 99) = 28.531, p < .0.001, $\eta_{p}^{2}$ = 0.587] and integrated power [F(4, 99) = 8.870, p<0.001, $\eta_{p}^{2}$ = 0.354]. Lower stride frequencies resulted in overall larger accelerations (Fig 2 and Table 1). Specifically, *post hoc* pairwise comparisons revealed that the lowest stride frequency (PSF-20%) yielded significantly larger magnitude and integrated power of the tibial accelerations than the other stride frequencies (p<0.01). No other significant differences were observed between any other of the stride conditions.

**Table 1:** Time, Frequency, and Shock Attenuation Characteristics of Tibial and Head Acceleration (Mean ± S.D).

	Visual Task		Stride Frequency				
	Free	Feedback	-20%	-10%	PSF	+10%	+20%
**Time Domain**							
**Tibia**							
**Impact acceleration peak (g) ^††^**	3.4600 (0.8193)	3.5371 (0.7980)	4.1937 (0.7786)	3.5485 (0.9707)	3.2521 (0.8246)	3.3071 (0.7538)	3.1916 (0.7154)
**Head**							
**Impact acceleration peak (g) ^††^**	1.2627 (0.3283)	1.2213 (0.3328)	1.4560 (0.4521)	1.2230 (0.3882)	1.1970 (0.2806)	1.1562 (0.2528)	1.1778 (0.2791)
Active acceleration peak (g) * ^††^	1.3624 (0.3057)	1.3116 (0.3129)	1.4914 (0.3616)	1.3994 (0.3460)	1.3529 (0.2855)	1.2679 (0.2856)	1.1733 (0.2679)
**Frequency Domain**							
**Tibia**							
Signal power magnitude (3~8 Hz) (g^2^ / Hz) * ^††^	0.1170 (0.0469)	0.1066 (0.0417)	0.1529 (0.0556)	0.1252 (0.0478)	0.1059 (0.0427)	0.0916 (0.0377)	0.0833 (0.0377)
**Signal power magnitude (9~20 Hz) (g^2^ / Hz) ^††^**	0.2382 (0.1202)	0.2445 (0.1470)	0.3121 (0.1873)	0.2438 (0.1356)	0.2194 (0.1187)	0.2130 (0.1188)	0.2184 (0.1077)
**Head**							
Signal power magnitude (3~8 Hz) (g^2^ / Hz) ** ^††^	0.1357 (0.0596)	0.1218 (0.0536)	0.1538 (0.0711)	0.1409 (0.0650)	0.1300 (0.0536)	0.1166 (0.0461)	0.1024 (0.0471)
**Signal power magnitude (9~20 Hz) (g^2^ / Hz) ^††^**	0.0383 (0.0208)	0.0347 (0.0181)	0.0462 (0.0394)	0.0332 (0.0194)	0.0319 (0.0132)	0.0326 (0.0114)	0.0383 (0.0139)
**Shock Attenuation**							
**Active Phase Magnitude (3~8 Hz) (dB) ^††^**	-3.7476 (9.7957)	-4.9953 (10.6990)	-7.0611 (11.0037)	-8.6680 (9.5481)	-7.1274 (10.1605)	-1.2641 (8.5960)	2.2632 (11.9285)
**Impact Phase Magnitude (9~20 Hz) (dB) ^††^**	-98.2031 (32.7374)	-98.1336 (32.1501)	-104.4268 (46.6673)	-107.0040 (30.7017)	-98.7252 (25.9558)	-93.8243 (33.1576)	-86.8615 (25.7365)

Main effect of Visual Task (* p < .05; ** p < .01). Main effect of Stride Frequency (^††^ p < .01). Positive values for Shock Attenuation indicate a gain in signal power whereas negative values indicate attenuation of signal power.

Main effects of stride frequency on the head accelerations during the impact portion of stance were observed for both the magnitude [F(4, 99) = 9.326, p < .0.001, $\eta_{p}^{2}$ = 0.342] and integrated power [F(4, 99) = 3.693, p<0.01, $\eta_{p}^{2}$ = 0.157] (Fig 2 and Table 1). Lower stride frequencies were related to larger magnitude and integrated power of head acceleration during the impact phase of stance. Specifically, during the impact portion of stance the magnitude of head acceleration in the PSF-20 condition differed from all other stride frequency conditions (p<0.001, for each comparison). No differences between the other stride frequency conditions were observed. The integrated power of the head acceleration in the PSF-20 differed from the PSF-10, PSF, and PSF+10 condition (p<0.01, for all conditions) but not from the PSF+20 (p = 0.078).

Main effects for stride frequency on the head acceleration during the active portion of stance were observed for the magnitude [F(4, 99) = 28.254, p < .0.001, $\eta_{p}^{2}$ = 0.648] and integrated power [F(4, 99) = 18.901, p<0.001, $\eta_{p}^{2}$ = 0.533] (Fig 2 and Table 1). Specifically, the magnitude of the head acceleration in the PSF-20 and PSF-10 conditions did not differ significantly (p>0.05), but were significantly larger than PSF, which was larger than PSF+10, which was again larger than PSF+20 (p<0.01 for all). Furthermore, during this same portion of stance the integrated power of the head accelerations while running at PSF-20, PSF-10, and PSF conditions did not differ, but these were all greater than PSF+10 which in turn was greater than PSF+20 (p<0.05, for all stated differences).

**Fig 3:**
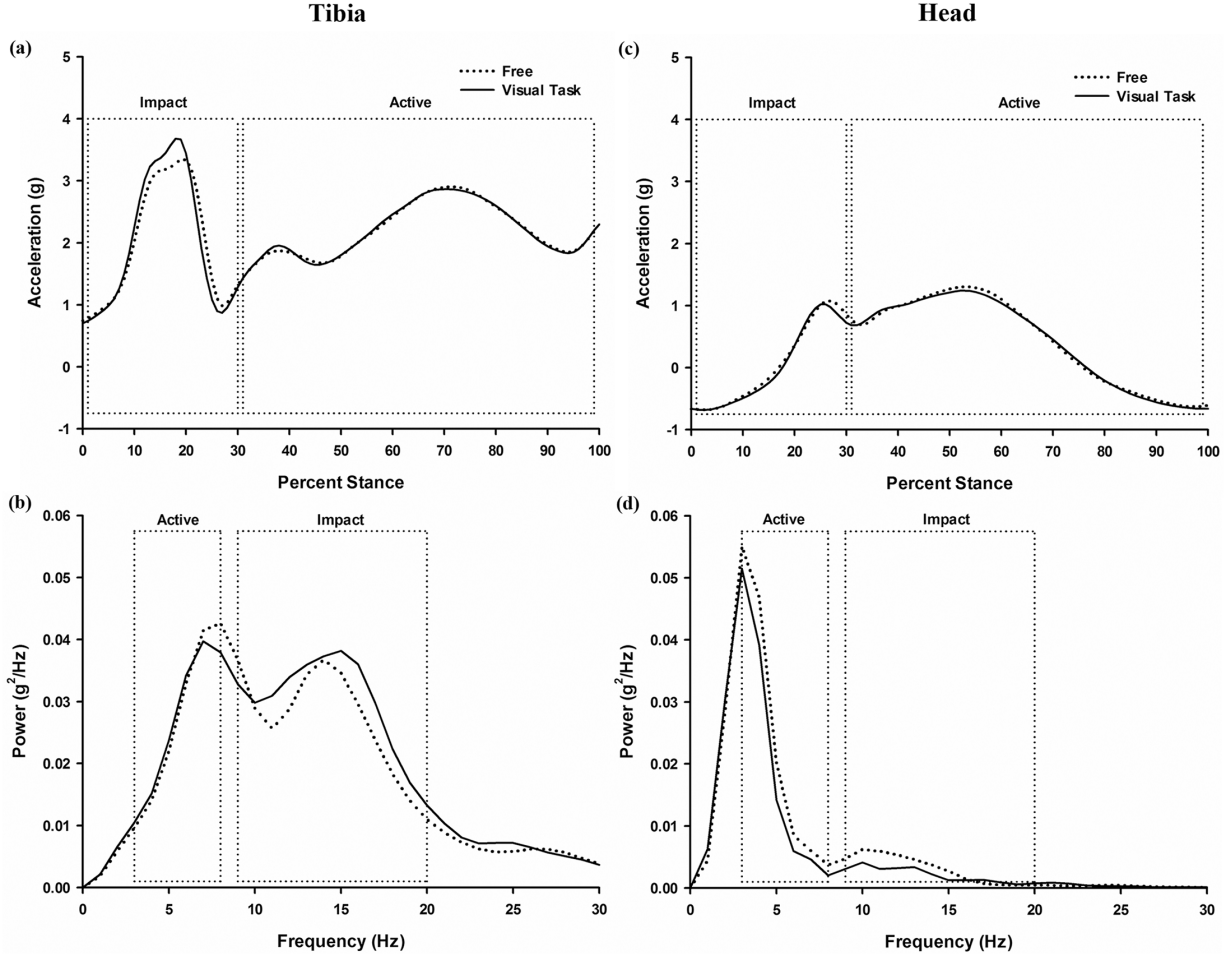
Tibial and head acceleration profiles during stance as a function of visual task. Representative participant data: tibia and head acceleration characteristic profile in time ((a) and (c)) and frequency ((b) and (d)) domain during a stance phase as a function of visual task.

No effect of visual task was observed for either the tibial or head peak accelerations and integrated power during the impact portion of stance (p>0.05) (Fig 3 and Table 1). However, there was a significant visual task effect on the peak head acceleration during the active portion of stance [F(1, 99) = 7.279, p<0.01, $\eta_{p}^{2}$ = 0.512], with lower head accelerations in the visual feedback condition (Fig 3 and Table 1). Additionally, a main effect of visual task was observed in the integrated power of the accelerations of both the tibia (F(1, 99) = 4.888, p<0.05, $\eta_{p}^{2}$ = 0.328) and the head (F(1, 99) = 11.293, p<0.001, $\eta_{p}^{2}$ = 0.575) during the active portion of stance (3–8 Hz).

**Fig 4:**
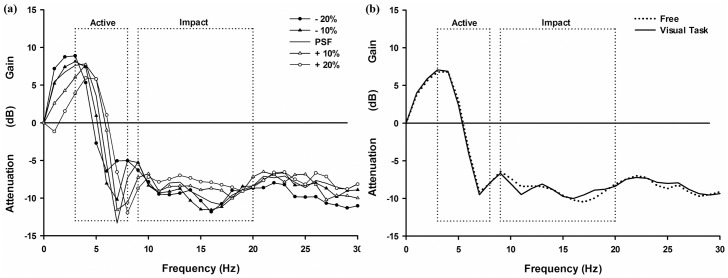
Transfer of shock from tibia to head as a function of stride frequency and visual task. Representative participant data: mean transfer functions between tibia and head accelerometer signals running at different frequency conditions (a) and visual task (b).

A main effect of stride frequency was observed in the transfer of shock from the tibia to the head in active [F(4, 99) = 8.770, p<0.001, $\eta_{p}^{2}$ = 0.411 at 3–8 Hz] and impact portions of stance [F(4, 99) = 3.752, p<0.01, $\eta_{p}^{2}$ = 0.165 at 9–20 Hz], such that lower stride frequency conditions were associated with greater attenuation of shock in both frequency ranges (Fig 4 and Table 1). Specifically, during both the active portion (3–8 Hz range) and impact portions of stance more shock was attenuated in the PSF-20, PSF-10, and PSF condition than in the PSF+10 and PSF+20 conditions (p<0.05, for all differences).

No effect of visual task condition was observed in the attenuation of shock from the tibia to the head during either the impact or active portions of stance phase of running (Fig 4 and Table 1).

### Head-Gaze Dynamics

**Fig 5:**
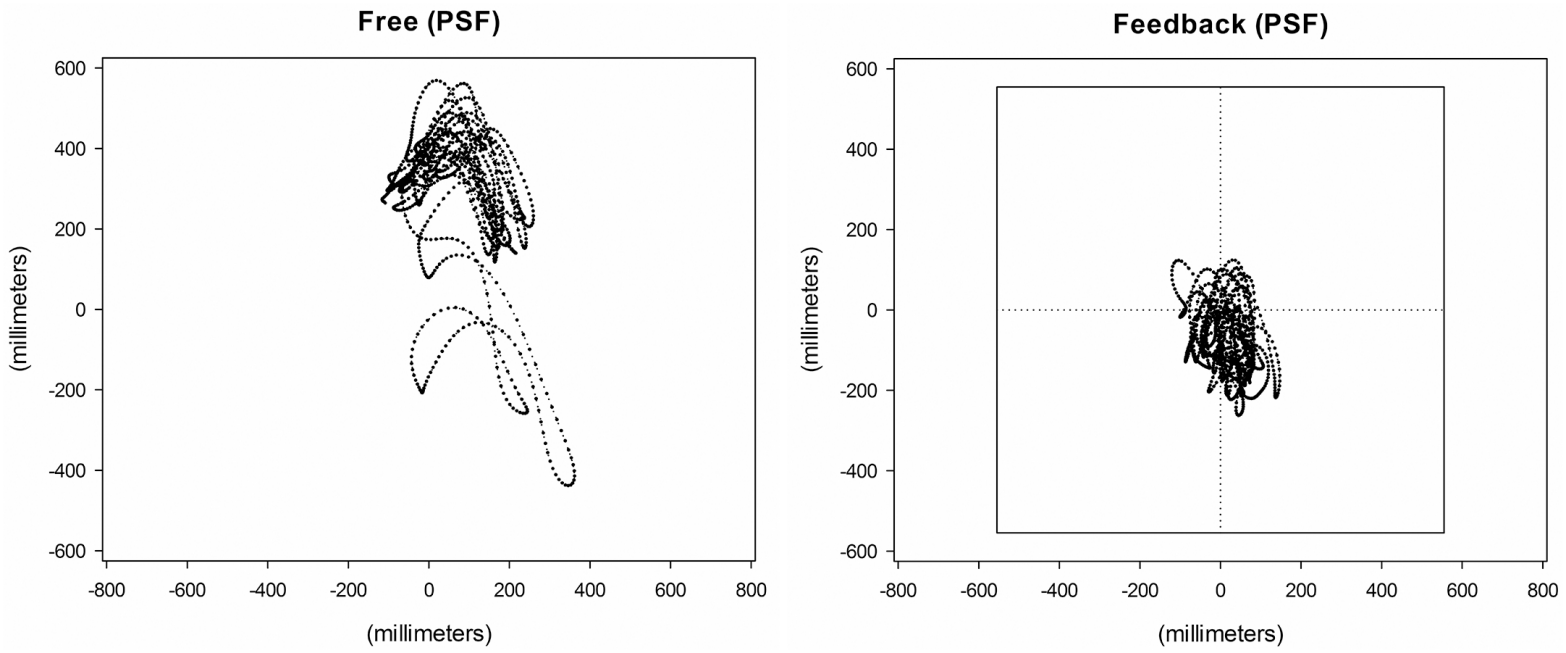
Head gaze trajectories during running. Reconstructed head gaze point trajectory of representative participant without (Left) and with (Right) visual feedback at preferred stride frequency (PSF).

Main effects for visual task condition were found such that, compared to the no visual feedback condition, in the visual feedback condition there was a reduction in the 95% ellipse area [F(1, 99) = 16.750, p<0.001, $\eta_{p}^{2}$ = 0.433], gaze path length [F(1, 99) = 88.234, p<0.001, $\eta_{p}^{2}$ = 0.772], mean velocity of the gaze path [F(1, 99) = 81.096, p<0.001, $\eta_{p}^{2}$ = 0.745], and range of motion of the gaze point in both the horizontal [F(1, 99) = 10.336, p<0.01, $\eta_{p}^{2}$ = 0.351] and vertical directions [F(1, 99) = 46.5522, p<0.001, $\eta_{p}^{2}$ = 0.751] (Fig 5 and Table 2).

**Table 2:** Head-gaze Point Dynamics as a Function of Visual Task and Stride Frequency (Mean ± S.D.).

	Visual Task	
	Free	Feedback
**Head-Gaze**		
95% ellipse area (cm^2^) **	54.381 (56.791)	18.124 (13.133)
Path-length resultant (mm) **	17623.0 (3753.4)	13588.6 (3905.5)
Mean velocity resultant (mm/sec^2^) **	1656.1 (322.6)	1291.9 (345.7)
Horizontal ROM (mm) **	730.7 (372.5)	517.3 (300.0)
Vertical ROM (mm) **	821.0 (438.4)	461.2 (159.5)

Main effect of Visual Task (* p < .05; ** p < .01).

## Discussion

The aim of this study was to examine the effect of providing visual feedback of head-gaze orientation on the magnitude, integrated power of tibial and head accelerations, the attenuation of shock from the tibia to the head, and head-gaze dynamics. The results support our first hypothesis that reducing stride frequency resulted in increased head and tibial accelerations during the impact portion of stance. However, pairwise *post hoc* comparisons revealed that it was only the very short frequency (longest stride) condition (PSF-20) that yielded significantly larger head and tibial accelerations than all the other conditions. Contrary to our prediction, providing individuals with feedback of their head-gaze orientation in the frontal visual plane did not alter the amount of shock attenuated through the kinematic chain. Finally, in support of our hypotheses, providing feedback of head-gaze orientation resulted in reductions in peak acceleration and power spectral density during the active (late) phase of stance, as well as overall magnitude of head-gaze dynamics.

The outcome of our stride frequency manipulation was as expected; systematically altering stride frequency resulted in higher impact accelerations at the tibia and a greater attenuation of high frequency—impact—shock in the low frequency stride conditions. These results are similar to those previously observed in the literature for this type of stride length/frequency manipulation at preferred speed [8, 10, 14, 15]. It appears that the primary mechanisms by which individuals tune the amount of shock dampened through the kinematic chain are the modulation of knee joint stiffness [7] and moment [8] as well as activation of muscles that span the knee [9]. We had similar findings to Hamill et al. [10], in that the changes in the amount of high-frequency impact shock attenuated through the kinematic chain yielded near constant head accelerations. The sole exception to this occurred at the lowest stride frequency condition (PSF-20) in which we observed significantly elevated impact peak head accelerations. These results suggest that individuals are able to tune the transmission of impact shock to the head across stride frequencies ranging from faster (PSF+20) to moderately slower (PSF-10) than preferred; however, during very low stride frequencies (PSF-20), individuals are unable to adequately compensate during the impact portion of stance. These changes in head stability occur at the same stride frequency condition (PSF-20) in which significant increases in the metabolic cost of transport are observed, while the other stride frequency conditions result in only small to moderate increases in the metabolic cost of transport [10, 18]. Collectively these findings indicate that recreational runners are unable to tune their running mechanics and energetics in a way that can accommodate this *extreme* stride frequency *condition*.

Though individuals are able to effectively modulate the amount of impact shock transmitted up the kinematic chain across stride frequencies ranging from 10% below to 20% above preferred, the results of this experiment also suggest that receiving feedback of their head-gaze orientation impacts how individuals control head motion while running [20]. The amount of shock attenuated through the kinematic chain did not change with the visual task in both impact and active phases of stance. The lack of difference in shock attenuation between the visual tasks appears to arise from systematic reductions in both the head and tibial accelerations during the active portion of stance. The observed changes in kinematics [7], kinetics [8], and muscle activity [9] are well suited to reduce high-frequency accelerations during impact, associated with reduced stride frequency, though additional changes also occur during late stance that appear to increase head stability throughout the entirety of stance. Beyond identifying how individuals alter running mechanics to stabilize their visual field while running at prescribed stride rates, further investigation is needed to identify how individuals would choose to freely alter their stride parameters (stride frequency/stride length) when running in conditions that place significant demands on visual information pick up and navigation of complex terrain.

The systematic reduction of head-gaze motion when individuals were provided with feedback of their head-gaze orientation supports Hamill et al.’s [10] suggestion that there is a perceptual underpinning to the regulation of the transfer of shock through the kinematic chain. Specifically, we observe here that individuals actively alter their head accelerations in late stance in order to reduce the magnitude and velocity of head-gaze while running at a range of stride frequencies. While the results of this study provide insight into some aspects of how individuals alter head motion during running, further investigation is needed to identify: 1) how individuals adjust gait parameters in response to visual task complexity; and 2) how individuals would choose to freely alter gait speed in order to co-optimize visual field stability and the metabolic cost of locomotion.

The changes in head accelerations and head-gaze performance as a function of the visual task suggest that individuals organize their running mechanics to stabilize the head and the visual field. The results of this study expand the knowledge of how individuals stabilize their head-gaze during running at preferred speed at prescribed stride frequencies. Specifically, when individuals are given feedback of their head-gaze position they appear to make adjustments in late stance that reduce head accelerations and overall degree of head movement. These changes in the regulation of head motion during locomotion likely play an important role in the how individuals are able to successfully navigate complex terrain [3–6, 20]. The pattern of the results indicate that lower head accelerations support reduced head-gaze motion (see Fig 5 and Tables 1 & 2), and that individuals would likely choose to increase stride frequency at any given speed to minimize head acceleration impact peaks when a high degree of visual stability is required.

In conclusion, the results of this study support previous findings that lower stride frequencies result in greater shock attenuation and a generalized stabilization of head accelerations [8, 10, 15]. Head and tibial accelerations resulting from initial foot-ground impact appear to be associated with stride frequency but not with the nature of the visual task imposed. The reduction of head acceleration during the active portion of stance when individuals are provided feedback of their head-gaze orientation appears to be the mechanism by which head-gaze motion is reduced. These results suggest that the control of head motion and the regulation of head accelerations during preferred speed is task dependent, such that individuals adopt movement patterns that reduce head accelerations and head-gaze motion.

## Supporting Information

S1 Data Complete data set. The spreadsheet contains all metrics for each subject for each condition.
(XLSX)
